# Expression, Prognosis, and Immune Infiltrates Analyses of E2Fs in Human Brain and CNS Cancer

**DOI:** 10.1155/2020/6281635

**Published:** 2020-12-15

**Authors:** Pan Liao, Shuzhen Han, Honglan Qu

**Affiliations:** ^1^Inner Mongolia University for the Nationalities, Tongliao, China; ^2^Department of Neurology, Inner Mongolia Forestry General Hospital, Yakeshi, Inner Mongolia, China; ^3^Department of Oncology, Inner Mongolia Forestry General Hospital, Yakeshi, Inner Mongolia, China

## Abstract

**Objective:**

We investigated the expression patterns, potential functions, unique prognostic value, and potential therapeutic targets of E2Fs in brain and CNS cancer and tumor-infiltrating immune cell microenvironments.

**Methods:**

We analyzed E2F mRNA expression levels in diverse cancer types via Oncomine and GEPIA databases, respectively. Moreover, we evaluated the prognostic values using GEPIA database and TCGAportal database and the correlation of E2F expression with immune infiltration and the correlation between immune cell infiltration and GBM and LGG prognosis via TIMER database. Then, cBioPortal, GeneMANIA, and DAVID databases were used for mutation analysis, PPI network analysis of coexpressed gene, and functional enrichment analysis.

**Results:**

E2F1-8 expression increased in most cancers, including brain and CNS cancer. Higher expression in E2F1, 2, 4, 6, 7, and 8 indicated poor OS of LGG. Higher E2F3–6 and E2F1–8 expressions correlated with poor prognosis and increased immune infiltration levels in CD8+ T cells, macrophages, neutrophils, and DCs in GBM and CD8+ T cells, B cells, CD4+ T cells, neutrophils, macrophages, and DCs in LGG, respectively.

**Conclusion:**

E2F1–8 and E2F2–8 could be hopeful prognostic biomarkers of GBM and LGG, respectively. E2F3–6 and E2F1–8 could be likely therapeutic targets in patients with immune cell infiltration of GBM and LGG, respectively.

## 1. Introduction

E2Fs, as a set of genes encoding transcription factor family (TFs) in higher eukaryotes, play an essential role in cell cycle regulation and DNA synthesis [1]. In some human malignancies, E2F activator expression is deactivated and is deregulated. A report demonstrating that increased expression in E2F3a played a significant role in the development of glioma. This study suggests that E2Fs could promote cancer development [2].

Worldwide, brain and CNS cancer are commonly diagnosed as cancers and the causes of tumor death [3]. Almost 80% of malignant brain cancers are gliomas [4]. In 2016, based on data from groundbreaking research [5], the WHO improved the correct classification of glioma subtypes by integrating tumor morphology and molecular genetic information [6]. The clinical therapy of these cancers includes surgery, radiotherapy, and chemotherapy, which is far from sufficient in combating cancer development. Tumor-infiltrating lymphocytes are an independent predictor of sentinel lymph node status, and survival in cancers [7] and immunotherapy has been considered a promising direction because of its ability to penetrate the blood-brain barrier to treat these tumors since the first discovery of lymphatic in CNS [8]. However, current immunotherapies, for example, anti-CTLA4, demonstrated poor clinical efficacy in brain tumors [9]. CAR T cells also still face substantial obstacles in treating brain tumors [10]. Besides, after the diagnosis of malignant brain and CNS tumors, the overall 5-year relative survival rate was 32.1%, of which the survival rate was only 4.9% after the diagnosis of GBM (glioblastoma multiforme) [4], and metastasis often leads to a poor prognosis. Due to the heterogeneity of tumors, currently, there are some limitations in the biomarkers that can predict prognosis, so it is necessary to search for new biomarkers in this field as prognostic indicators, to improve prognosis. Also, there is an urgent need to identify new therapeutic targets in the brain and CNS cancer so as to effectively improve the accuracy of treatment.

E2F plays a complex and unique role in human brain and CNS tumors. E2F1/DP1 complex was reported to inhibit the mismatch repair proteins MSH2, MSH6, and EXO1, as well as the homologous recombinant protein RAD51, leading to cell apoptosis [11]. Overexpression of E2F1 blocked the proliferation repression caused by miR-342-3p or miR-377 in glioma cells [12], and E2F1 overexpression conferred resistance to cisplatin in glioma cells [13]. Studies have shown that downregulation of E2F2 inhibits glioma cell growth [14]. Overexpression of E2F2 promoted the progression of NSCLC cells [15]. Increased expression in E2F3a played a significant function in glioma development [2]. Change in human GBM cell cycle is related to the decrease of E2F4 level [16]. The silencing of E2F5 inhibited the proliferative ability of GBM cells [17]. Moreover, the combination of E2F6 and MATN1-AS1, which negatively targeted RELA, inhibited the MAPK signaling pathway, thus inhibiting the proliferation and invasion of GBM cells [18]. E2F7 could induce autophagy of glioma [19]. Targeting E2F8 can also regulate the development in glioma [20]. However, the underlying mechanisms of E2F activation or inhibition and the distinct functions of E2Fs and tumor immunology in brain and CNS tumor have not been fully elucidated.

As far as we know, bioinformatics analysis and immune infiltrate analysis have not been used to study the function of E2Fs from tumors of the brain and CNS. As an important part of biological and biomedical research, RNA and DNA research has undergone revolutionary changes due to microarray technology [21]. In addition, immunotherapy, a revolution in cancer treatment, has become a promising strategy [22]. Based on thousands of gene expression or copy number variations published online, we analyzed the expression patterns, potential functions, and prognostic value of E2Fs in brain and CNS cancer. Furthermore, we analyzed the interrelationship between E2Fs and immune cell in different cancer microenvironments to identify underlying therapeutic targets of brain and CNS tumor.

## 2. Materials and Methods

### 2.1. GEPIA Dataset Analysis

GEPIA was used to analyze the RNA sequencing expression data from 9,736 tumors and 8,587 normal samples from TCGA and the Genotype-Tissue Expression (GTEx) projects. GEPIA has many functional modules, including differential expression, survival, and correlation analysis [23].

### 2.2. Oncomine Dataset Analysis

The transcription levels of E2Fs of various tumors and coexpression genes of E2Fs in brain and CNS cancer were determined through analysis in Oncomine cancer microarray datasets.

### 2.3. The Cancer Genome Atlas Data [24] and cBioPortal Datasets Analyses

cBioPortal currently contains 225 cancer studies to explore multidimensional tumor genomics data sets [25]. We used cBioPortal to analyze E2F alterations in the glioblastoma multiforme (TCGA, Firehose Legacy) dataset, including data from 604 cases with pathology reports, and the brain lower-grade glioma (TCGA, PanCancer Atlas) dataset, including data from 514 cases with pathology reports. The search parameters included mutations, putative copy number alterations (CNAs) from genomic identification of significant targets in cancer (GISTIC), mRNA expression *Z* scores (RNA-seq v.2 RSEM), and protein expression *Z* scores (reverse phase protein array (RPPA)). Additionally, Kaplan–Meier survival curves were drawn in cBioPortal to evaluate the influence of gene alterations of E2Fs on the overall survival of GBM and LGG patients.

### 2.4. TCGAportal Database Analysis

TCGAportal was used to further verify the prognostic value of mRNA level of E2F factors in GBM and LGG patients.

### 2.5. TIMER Database Analysis

TIMER database includes 10,897 samples across 32 cancer types from The Cancer Genome Atlas (TCGA) to estimate the abundance of immune infiltrates [26]. We analyzed the correlation of the E2F expression with the abundance of immune infiltrates, including B cells, CD4+ T cells, CD8+ T cells, neutrophils, macrophages, and dendritic cells, via gene modules. In addition, correlations between immune cell infiltration and GBM and LGG prognosis were explored via correlation modules. Lastly, we assessed how the E2F expression correlated with the expression of particular immune infiltrating cell subset markers.

### 2.6. GeneMANIA Database Analysis

GeneMANIA is a flexible web to construct protein-protein interaction networks, generating hypotheses on gene role, exploring gene list, while prioritizing genes [27]. We visualized the gene network and predicted the functions of E2F coexpression genes via GeneMANIA.

## 3. Results

### 3.1. Expression Levels of E2Fs of Brain and CNS Tumor

**Figure 1:**
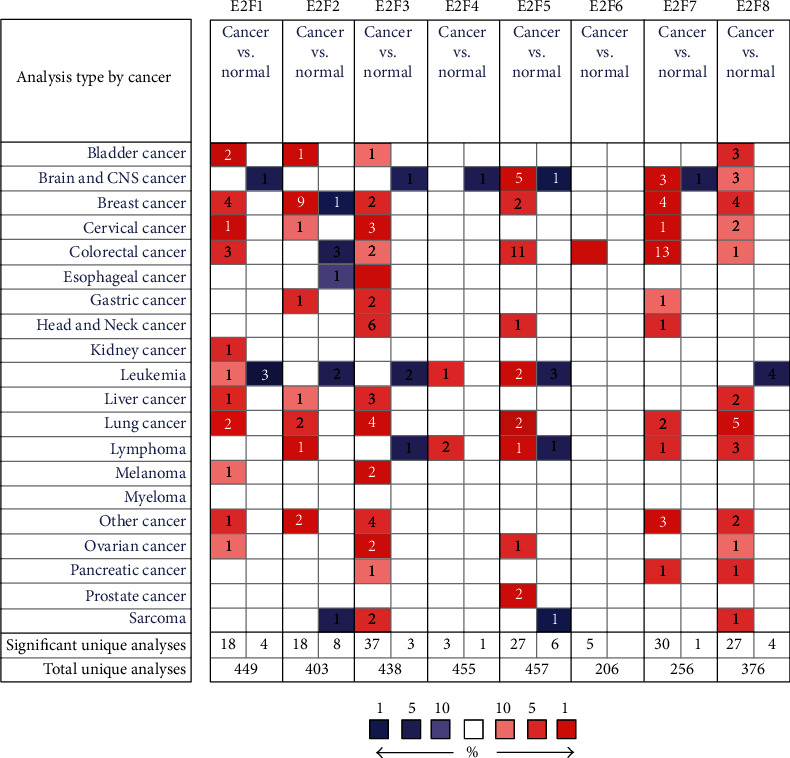
The transcription levels of E2F factors in different types of cancers (Oncomine).

Oncomine differential expression analysis revealed that the E2F5 expression was remarkably upregulated in brain and CNS tumor in five datasets (Figure 1).

In the French Brain Statistics [28], E2F5 was overexpressed in anaplastic oligodendroglioma versus normal tissues with a fold change of 2.425 (Table 1). In the Sun Brain Statistics (Table 1) [29], E2F5 was also overexpressed in anaplastic astrocytoma with a fold change of 2.545, in oligodendroglioma with a fold change of 2.580, and in glioblastoma with a fold change of 2.877. In the Murat Brain Statistics (Table 1) [30], E2F5 was also overexpressed in glioblastoma with a fold change of 2.016. The Murat Brain Statistics (Table 1) [30] showed that E2F7 was also increased in glioblastoma (fold change = 4.632) compared to normal samples. In addition, in the Sun Brain Statistics (Table 1) [29], E2F7 was overexpressed in glioblastoma (fold change = 5.823), and in the French Brain Statistics (Table 1) [28], the increased E2F7 expression was found in anaplastic oligodendroglioma compared to normal sample (fold change = 2.282). In the Sun Brain Statistics (Table 1) [29], E2F8 was also overexpressed in anaplastic astrocytoma (fold change = 4.196), glioblastoma (fold change = 7.097), and oligodendroglioma (fold change = 3.341) compared to normal samples.

**Figure 2:**
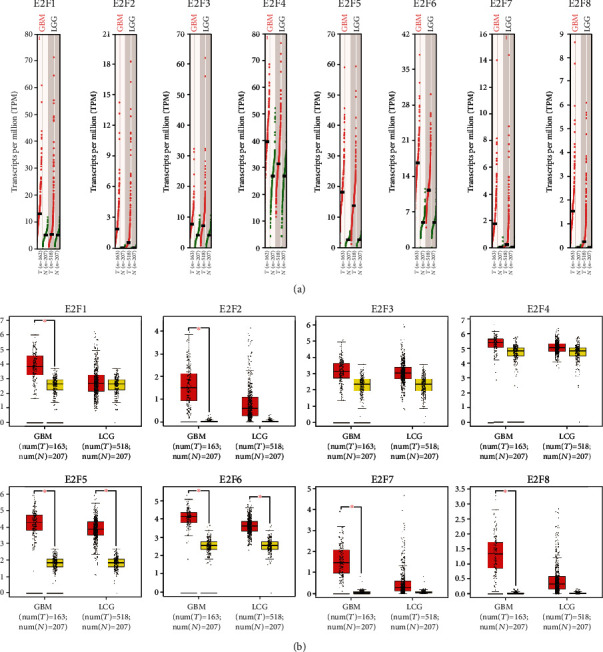
The expression of E2Fs in GBM and LGG (GEPIA): (a) scatter diagram; (b) box plot.

GEPIA differential expression analysis indicated that the E2F1-8 expressions were higher in GBM tissue compared with normal tissue and that E2F2-8 was higher in LGG (Figure 2).

### 3.2. The Correlations between Different E2Fs in Brain and CNS Cancer

**Figure 3:**
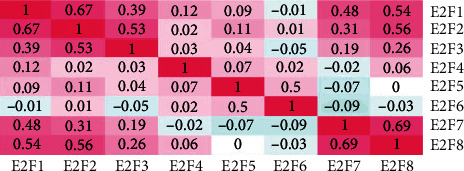
The correction between different E2Fs in brain and CNS cancer (cBioPortal).

The cBioPortal online tool calculated Pearson correlations. The results showed that the following E2Fs showed significantly positive correlations: E2F1 with E2F2, E2F3, E2F7, and E2F8; E2F2 with E2F1, E2F3, E2F7, and E2F8; E2F3 with E2F1 and E2F2; E2F7 with E2F1, E2F2, and E2F8; and E2F8 with E2F1, E2F2, and E2F7 (Figure 3).

### 3.3. Gene Alteration of E2Fs in Brain and CNS Cancer Tissue from cBioPortal

**Figure 4:**
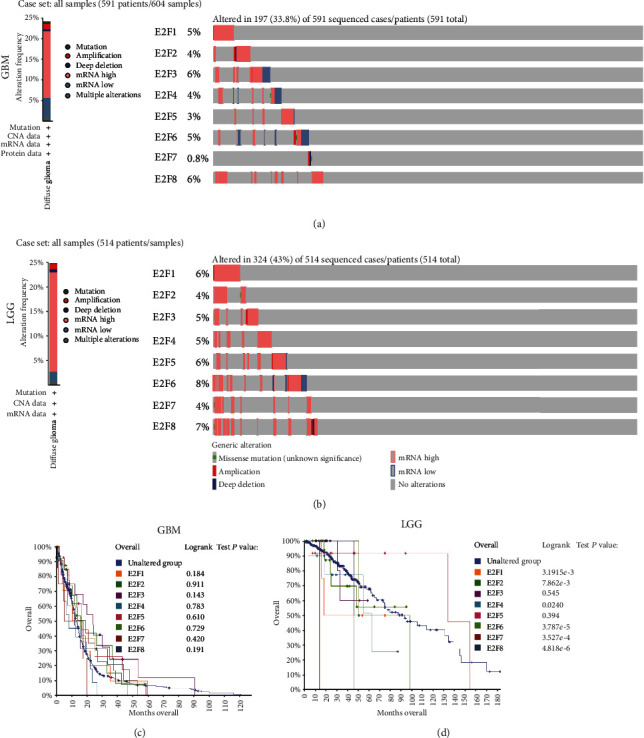
E2F gene expression, mutation analysis, and survival analysis in brain and CNS cancer (cBioPortal): (a) E2F gene expression and mutation analysis in GBM; (b) E2F gene expression and mutation analysis in LGG; (c) overall survival analysis for gene alteration of E2Fs in GBM patients; (d) overall survival analysis for gene alteration of E2Fs in LGG patients.

cBioPortal analyses revealed that E2Fs were altered in 197 (33·8%) of all the 591 sequenced cases with GBM, which included 3 mutation, 8 cases of amplification, 3 cases of deep deletion, 29 cases of mRNA downregulation, 97 cases of mRNA upregulation, and 5 cases of multiple alterations. Statistical results of the gene alteration frequency indicated that mRNA upregulation occupied the overwhelming majority of alteration types (Figure 4(a)). E2Fs were altered in 324 (43%) of all the 514 sequenced cases with LGG, which included 1 mutation, 6 cases of amplification, 3 cases of deep deletion, 10 cases of mRNA downregulation, 103 cases of mRNA upregulation, and 4 cases of multiple alterations. Among the LGG cases with gene alteration of E2Fs, mRNA upregulation occupied the overwhelming majority of alteration types (Figure 4(b)). Kaplan–Meier overall survival curves showed that gene alterations of E2F1, E2F2, E2F4, and E2F6-8 were remarkably related to overall survival (OS) (*P* < 0.05) among LGG (Figure 4(d)). No significant correlation was identified between gene alteration of E2Fs and OS in GBM patients (Figure 4(c)).

### 3.4. The Prognostic Value of E2Fs in GBM and LGG Patients

**Figure 5:**
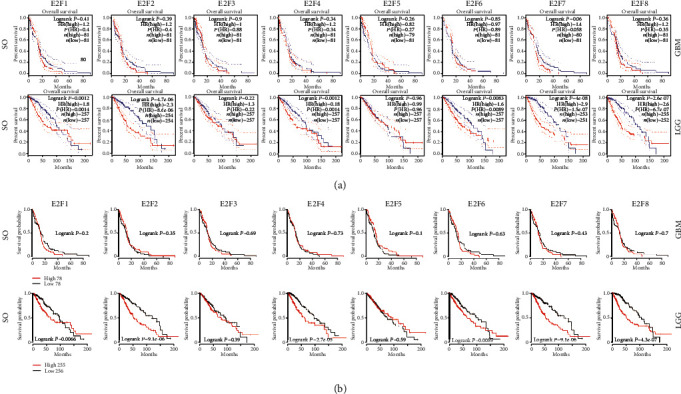
The prognostic value of mRNA level of E2F factors in GBM and LGG patients: (a) the prognostic value of mRNA level of E2F factors in GBM and LGG patients (GEPIA); (b) the prognostic value of mRNA level of E2F factors in GBM and LGG patients (TCGAportal).

GEPIA and TCGAportal survival analysis both revealed that decreased E2F1, E2F2, E2F4, and E2F6-8 mRNA levels were remarkably related to overall survival (OS) (*P* < 0.05) among LGG. No significant correlation was identified between E2F mRNA levels and OS in GBM patients (Figure 5).

### 3.5. Prognostic Relevance of Immune Cell Infiltration in GBM and LGG

**Figure 6:**
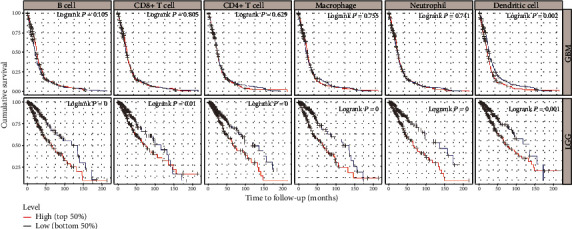
Immune cell infiltration survival curve of GBM and LGG patients (TIMER). Red indicates a high degree of infiltration, and blue indicates a low degree of infiltration. *P* < 0.05 was considered significant, and *P* < 0.0001 was represented by 0.

TIMER analysis showed that the survival time of LGG patients with low B cells, macrophage, CD4+ T cells, neutrophil, and DCs infiltrations was significantly higher than that of LGG patients with high infiltration. However, there were no meaningful findings about GBM (Figure 6).

### 3.6. Correlations of E2F Expression with Immune Infiltration Levels in GBM and LGG

**Figure 7:**
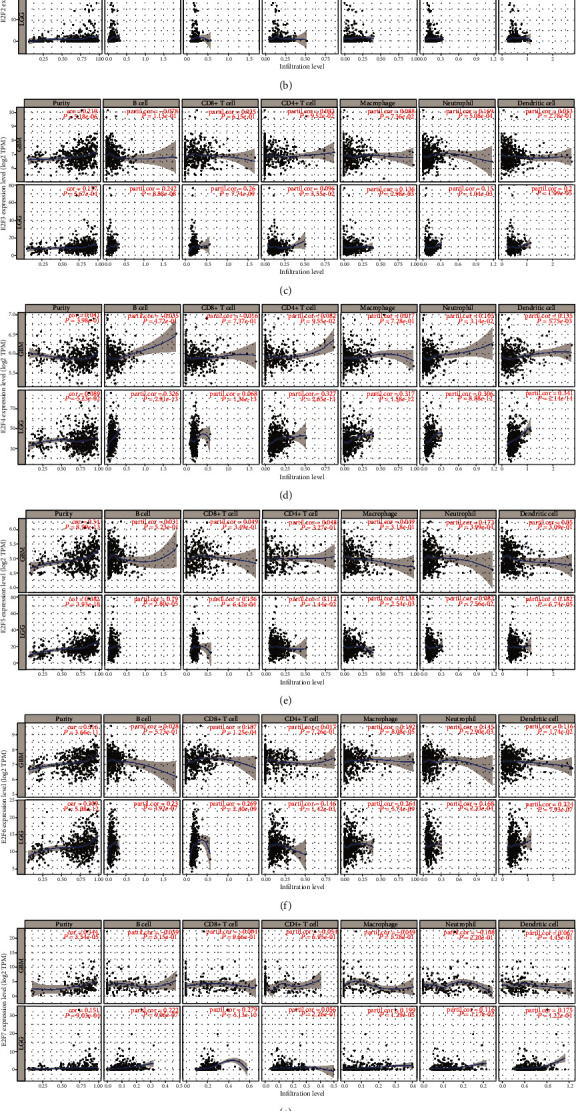
Correlation of the E2F expression with immune infiltration level in GBM and LGG: (a) correlation of the E2F1 expression with immune infiltration level in GBM and LGG; (b) correlation of the E2F2 expression with immune infiltration level in GBM and LGG; (c) correlation of the E2F3 expression with immune infiltration level in GBM and LGG; (d) correlation of the E2F4 expression with immune infiltration level in GBM and LGG; (e) correlation of the E2F5 expression with immune infiltration level in GBM and LGG; (f) correlation of the E2F6 expression with immune infiltration level in GBM and LGG; (g) correlation of the E2F7 expression with immune infiltration level in GBM and LGG; (h) correlation of the E2F8 expression with immune infiltration level in GBM and LGG.

TIMER analysis revealed that the E2F1 expression is remarkably positively related to tumor purity (*r* = 0.347, *P* = 2.46*e* − 13) in GBM (Figure 7(a)). And the E2F1 expression demonstrated a very weak interrelation with CD8+ T cell infiltration levels in LGG (*r* = 0.126, *P* = 5.76*e* − 03). Also, the E2F2 expression was significantly positively related to cancer purity (*r* = 0.172, *P* = 4.09*e* − 04) and remarkably negatively related to macrophages (*r* = −0.183, *P* = 1.65*e* − 04), neutrophils (*r* = −0.305, *P* = 1.88*e* − 10), and dendritic cells (*r* = −0.264, *P* = 4.23*e* − 08) but no significant correlation with CD4+ T cells in GBM (Figure 7(b)). E2F2 expression level was remarkably positively related to infiltrating levels of B cells (*r* = 0.359, *P* = 5.53*e* − 16), CD4+ T cells (*r* = 0.339, *P* = 3.13*e* − 14), macrophages (*r* = 0.285, *P* = 2.63*e* − 10), neutrophils (*r* = 0.293, *P* = 7.29*e* − 11), and dendritic cells (*r* = 0.371, *P* = 6.15*e* − 17) but no significant correlation with tumor purity (*r* = 0.119, *P* = 8.88*e* − 03) in LGG (Figure 7(b)). The E2F3 expression was remarkably positively related to tumor purity (*r* = 0.219, *P* = 5.81*e* − 06) and neutrophils (*r* = 0.293, *P* = 7.29*e* − 11) in GBM (Figure 7(c)). The E2F3 expression was remarkably positively related to tumor purity (*r* = 0.157, *P* = 5.67*e* − 04) and infiltrating levels of B cells (*r* = 0.242, *P* = 8.88*e* − 08), CD8+ T cells (*r* = 0.26, *P* = 7.74*e* − 09), and DCs (*r* = 0.2, *P* = 1.09*e* − 05) but no significant correlation with macrophages (*r* = 0.136, *P* = 2.98*e* − 03) or neutrophils (*r* = 0.15, *P* = 1.04*e* − 03) in LGG (Figure 7(c)). The E2F4 expression demonstrated a very weak interrelation with dendritic cell (*r* = 0.135, *P* = 5.75*e* − 03) infiltration levels in GBM (*r* = 0.126, *P* = 5.76*e* − 03). The E2F4 expression was remarkably positively related to infiltrating levels of B cells (*r* = 0.326, *P* = 2.91*e* − 13), CD4+ T cells (*r* = 0.327, *P* = 2.65*e* − 13), macrophages (*r* = 0.317, *P* = 1.58*e* − 12), neutrophils (*r* = 0.306, *P* = 8.88*e* − 12), and dendritic cells (*r* = 0.341, *P* = 2.14*e* − 14) in LGG (Figure 7(d)). The E2F5 expression was remarkably positively related to tumor purity (*r* = 0.34, *P* = 8.99*e* − 13) and neutrophils (*r* = 0.172, *P* = 3.99*e* − 04) in GBM (Figure 7(e)). The E2F5 expression was remarkably positively related to tumor purity (*r* = 0.382, *P* = 3.93*e* − 18), B cells (*r* = 0.19, *P* = 2.80*e* − 05), CD8+ T cells (*r* = 0.156, *P* = 6.42*e* − 04), and DCs (*r* = 0.182, *P* = 6.74*e* − 05) but no significant correlation with macrophages (*r* = 0.138, *P* = 2.54*e* − 03) in LGG (Figure 7(e)). The E2F6 expression was remarkably positively related to tumor purity (*r* = 0.316, *P* = 3.66*e* − 11), CD8+ T cells (*r* = 0.187, *P* = 1.25*e* − 04), and macrophages (*r* = 0.192, *P* = 8.08*e* − 05) but no significant correlation with neutrophils (*r* = 0.145, *P* = 2.90*e* − 03) in GBM (Figure 7(f)). The E2F6 expression was remarkably positively related to tumor purity (*r* = 0.309, *P* = 5.08*e* − 12), B cells (*r* = 0.23, *P* = 3.92*e* − 07), macrophages (*r* = 0.264, *P* = 5.74*e* − 09), CD8+ T cells (*r* = 0.269, *P* = 2.40*e* − 09), neutrophils (*r* = 0.168, *P* = 2.33*e* − 04), and DCs (*r* = 0.224, *P* = 7.93*e* − 07) but no significant correlation with CD4+ T cells in LGG (Figure 7(f)). The E2F7 expression was remarkably positively related to tumor purity (*r* = 0.344, *P* = 3.54*e* − 05) in GBM (Figure 7(g)). The E2F7 expression was remarkably positively related to tumor purity (*r* = 0.151, *P* = 9.03*e* − 04), B cells (*r* = 0.222, *P* = 9.06*e* − 07), CD8+ T cells (*r* = 0.279, *P* = 5.13*e* − 10), macrophages (*r* = 0.199, *P* = 1.29*e* − 05), and DCs (*r* = 0.175, *P* = 1.22*e* − 04) in LGG (Figure 7(g)). The E2F8 expression was remarkably positively related to tumor purity (*r* = 0.335, *P* = 2.00*e* − 12) in GBM. The E2F8 expression was remarkably positively related to infiltrating levels of B cells (*r* = 0.283, *P* = 2.85*e* − 10), CD8+ T cells (*r* = 0.228, *P* = 4.48*e* − 07), CD4+ T cells (*r* = 0.218, *P* = 1.57*e* − 06), macrophages (*r* = 0.323, *P* = 6.23*e* − 13), neutrophils (*r* = 0.228, *P* = 5.00*e* − 07), and dendritic cells (*r* = 0.288, *P* = 1.64*e* − 10) in LGG (Figure 7(h)).

### 3.7. Assessment of the Correlation between E2Fs and Immune Marker Expression

In particular, TIMER analysis revealed that E2F1 was significantly correlated with monocyte markers (CD86), TAM markers (CCL2, IL10), and M2 macrophage markers (VSIG4, MS4A4A) in GBM (*P* < 0.0001; Table 2). E2F2 was significantly correlated with monocyte markers (CD86), TAM markers (CCL2, IL10), and M2 macrophage markers (VSIG4) in GBM (*P* < 0.0001; Table 2). E2F5 was significantly correlated with monocyte markers (CD115) and M1 macrophage markers (IRF5) in GBM (*P* < 0.0001; Table 2). E2F6 was significantly correlated with monocyte markers (CD86, CD115), TAM markers (CD68, IL10), M1 macrophage markers (IRF5), and M2 macrophage markers (VSIG4, MS4A4A) in GBM (*P* < 0.0001; Table 2). E2F7 was significantly correlated with TAM markers (IL10) in GBM (*P* < 0.0001; Table 2). E2F8 was significantly correlated with M2 macrophage markers (VSIG4) in GBM (*P* < 0.0001; Table 2). Meanwhile, E2F1 was significantly correlated with monocyte markers (CD86, CD115), TAM markers (CCL2), M1 macrophage markers (INOS), and M2 macrophage markers (VSIG4) in LGG (*P* < 0.0001; Table 2). E2F2 was significantly correlated with monocyte markers (CD86, CD115), TAM markers (CD68, IL10), M1 macrophage markers (IRF5), and M2 macrophage markers (CD163, VSIG4, MS4A4A) in LGG (*P* < 0.0001; Table 2). E2F4 was significantly correlated with monocyte markers (CD86), TAM markers (CD68), M1 macrophage markers (IRF5), and M2 macrophage markers (CD163, VSIG4, MS4A4A) in LGG (*P* < 0.0001; Table 2). E2F8 was significantly correlated with monocyte markers (CD68), TAM markers (CCL2, IL10), M1 macrophage markers (IRF5), and M2 macrophage markers (CD163, MS4A4A) in LGG (*P* < 0.0001; Table 2).

### 3.8. Functional Enrichment Analysis of Coexpressed Genes Correlated with E2Fs in Brain and CNS Cancer

**Figure 8:**
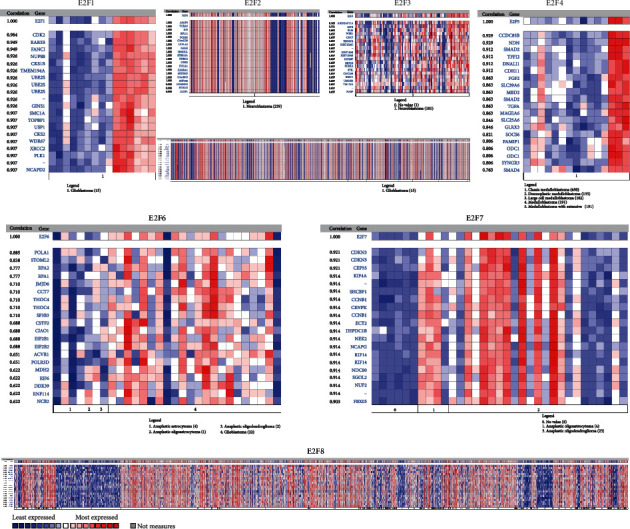
Coexpressed genes of E2Fs in brain and CNS cancer (Oncomine).

Oncomine analyses revealed that E2F1 was positively related to CDK2, RAB3B, FANCI, NUP88, CKS1B, TMEM194A, UBE2S, GINS1, SMC1A, TOPBP1, and USP1. E2F2 was positively corrected with ASAP3, TCEA3, ID3, RPL11, TCEB3, C1orf128, LYPLA2, GALE, HMGCL, SFRS13A, and PNRC2. E2F3 was positively corrected with ARHGAP11A, MYB, BLM, WEE1, CDC7, EXOSC8, HIST1H4C, HIST1H4I, HIST1H4B, IGF2BP, and RRP1B. E2F4 was positively corrected with ELMO3, LRRC29, EXOC3L, KIAA0895L, NOL3, HSF4, FBXL8, TRADD, B3GNT9, FHOD1, and TMEM208. E2F5 was positively corrected with CCDC85B, NDN, SMAD2, TFPI2, DNALI1, CDH11, FGF2, SLC39A6, MBD2, SMAD2, and TGFA. E2F6 was positively corrected with POLA1, STOML2, RPA2, RPA1, JMJD6, CCT7, THOC4, SF3B3, CSTF2, CIAO1, and EIF2B1. E2F7 was positively corrected with CDKN3, CEP55, KIF4A, SHCBP1, CCNB1, CENPK, ECT2, DEPDC1B, NEK2, NCAPG, and KIF14. E2F8 was positively corrected with KIFC1, CENPM, MYBL2, ERCC6L, AURKB, TK1, MCM10, CDCA3, NCAPH, NUSAP1, and MKI67 (Figure 8).

**Figure 9:**
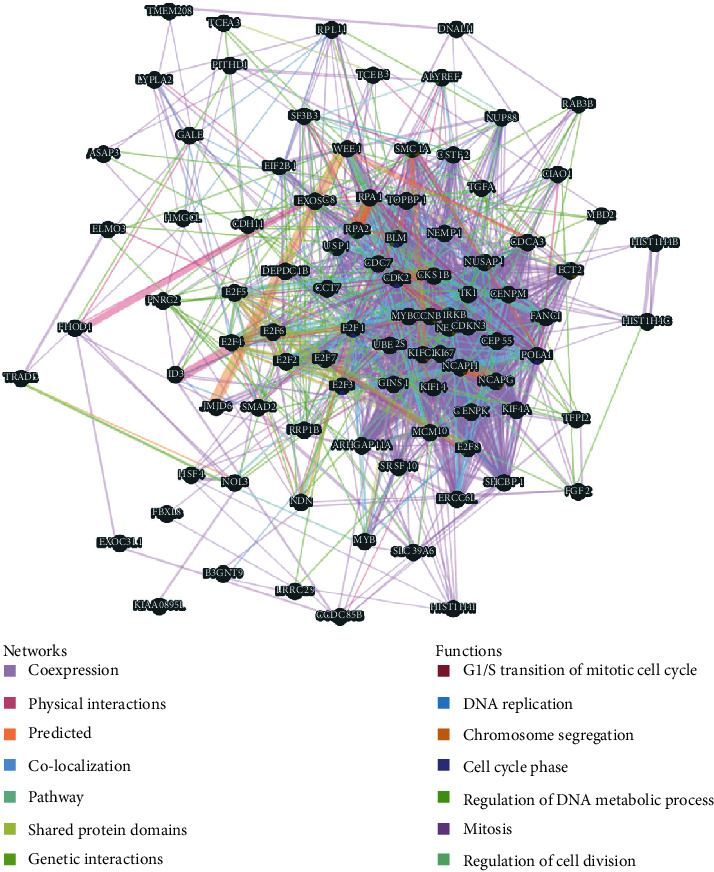
Protein-protein interaction network of E2F coexpression genes (GeneMANIA). Different colors of the network edge indicate the bioinformatics methods applied: coexpression, website prediction, pathway, physical interactions, and colocalization. The different colors for the network nodes indicate the biological functions of the set of enrichment genes.

Next, we constructed a network of E2Fs and E2F-correlated coexpressed genes via GeneMANIA. The results showed that the biological functions of the enriched gene set, including DNA replication, chromosome segregation, mitosis, regulation of cell division, cell cycle phase, and regulation of DNA metabolic process, were intimately associated with E2F alterations (Figure 9).

**Figure 10:**
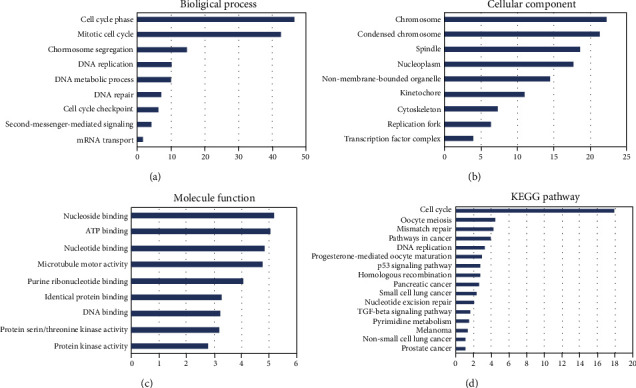
Significantly enriched GO annotations and KEGG pathways of E2Fs in brain and CNS cancer (DAVID). The functions of E2Fs and E2F coexpression genes were predicted by the analysis of gene ontology (GO) and Kyoto Encyclopedia of Genes and Genomes (KEGG) by DAVID (Database for Annotation, Visualization, and Integrated Discovery) tools (https://david.ncifcrf.gov/summary.jsp): (a) cellular components; (b) biological processes; (c) molecular functions; (d) KEGG pathway analysis.

The GO (gene ontology) and KEGG pathways in the DAVID database were analyzed to predict the functions of E2Fs and E2F-correlated coexpressed genes. Based on biological processes, cell components, and molecular functions, GO enrichment analyses predicted the functions of target host genes. We found that cell cycle phase, mitotic cell cycle, DNA replication, cell cycle checkpoint, and mRNA transport were remarkably regulated by E2F alterations in brain and CNS cancer (Figure 10(a)). Chromosome, replication, transcription factor complex, and protein serine/threonine kinase activity were also significantly controlled by these E2F alterations (Figures 10(b) and 10(c)). The corresponding genes are known to be associated with cell cycle.

**Figure 11:**
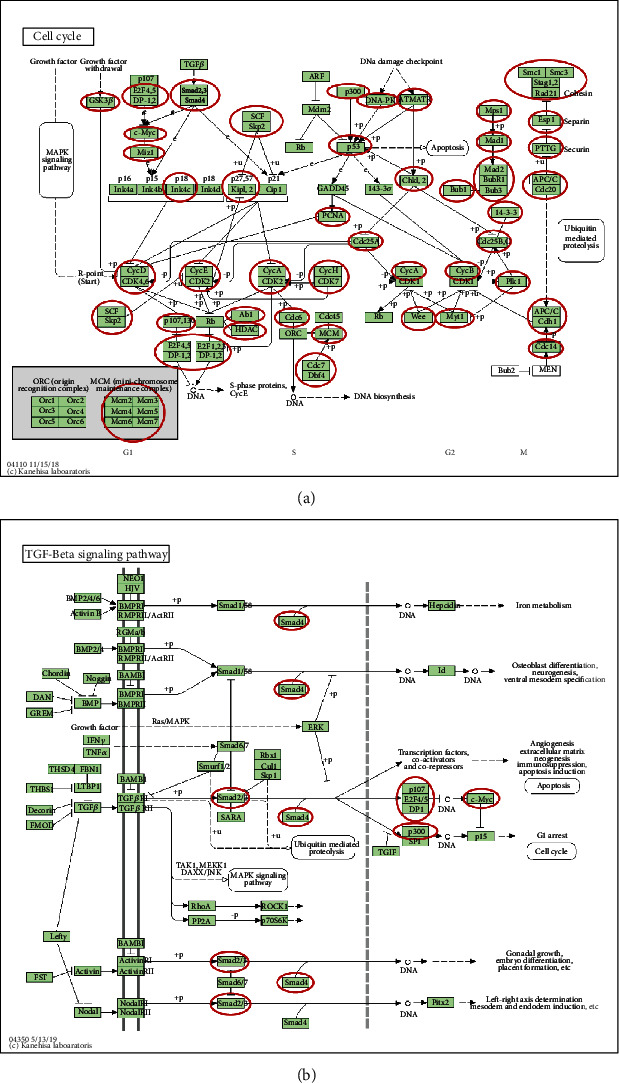
(a) Cell cycle and (b) TGF-beta-signaling pathway regulated by the E2F alteration in brain and CNS cancer.

KEGG analysis can define the pathways related to the functions of E2F alterations and coexpressed genes correlated with E2Fs. Nine pathways related to the functions of E2F alterations in brain and CNS cancer were found via KEGG analysis (Figure 10(d)). Among these pathways, cfa04110: cell cycle, cfa04350: TGF-beta signaling pathway, ptr05200: pathways in cancer, and cfa04115: p53 signaling pathway were involved in the tumorigenesis and pathogenesis about brain and CNS cancer (Figures 11(a) and 11(b)).

## 4. Discussion

The function of E2F activators in tumorigenesis and their prognostic relevance has been partially demonstrated in several cancers [31–33]; however, further bioinformatics analysis and analysis of immune infiltrates in brain and CNS cancers have not been performed. This is the first study that explored the mRNA expressions of different E2F factors in brain and CNS cancer and investigated their prognostic relevance and correlation with various tumor-infiltrating immune cells. Our findings may help improve treatment of patients with brain and CNS cancers.

E2F1 has been studied extensively in tumors of the brain and CNS [11–13, 34–38]. E2F1/DP1 complex was reported to inhibit the mismatch repair proteins MSH2, MSH6, and EXO1, as well as the homologous recombinant protein RAD51, leading to cell apoptosis [11]. Overexpression of E2F1 blocked the proliferation repression caused by miR-342-3p or miR-377 in glioma cells [12], and E2F1 overexpression conferred resistance to cisplatin in glioma cells [13]. MicroRNA-138 inhibited the development of GBM by targeting E2F1 [34]. PPAR*α* inhibited growth of glioma cells through the E2F1/miR-19a feedback loop [35]. miRNA-320 and miRNA-329 inhibited glioma proliferation by targeting E2F1 [36, 37]. The ECT2/PSMD14/PTTG1 axis promoted glioma proliferation by stabilizing E2F1 [38]. In this study, analysis of Oncomine dataset and GEPIA dataset revealed that elevated expression of E2F1 in human GBM. Analysis of GEPIA and TCGAportal datasets revealed the prognostic value of E2F1 in patients with GBM and LGG. High E2F1 expression and gene alteration of E2F1 in LGG was associated with poor OS; however, the E2F1 expression and gene alteration of E2F1 in GBM showed no significant correlation with OS. In TIMER datasets, the E2F1 expression in GBM showed a positive association with tumor purity while the E2F1 expression in LGG demonstrated a very weak interrelation with CD8+ T cells infiltration level. Specifically, E2F1 was significantly correlated with monocyte markers (CD86), TAM markers (CCL2, IL10), and M2 macrophage markers (VSIG4, MS4A4A) in GBM. E2F1 was significantly correlated with monocyte markers (CD86, CD115), TAM markers (CCL2), M1 macrophage markers (INOS), and M2 macrophage markers (VSIG4) in LGG. This suggests that E2F1 plays a role in regulating monocyte polarization in GBM and LGG.

Downregulation of E2F2 was shown to inhibit glioma cell growth in vitro and in vivo by inducing cell cycle arrest in G0/G1 [14]. Overexpression of E2F2 was found to promote invasive growth of NSCLC cells [15]. miR-125b regulated glioblastoma progress via E2F2 [39]. In our study, we observed overexpression of E2F2 in GBM and LGG tissues. However, while high E2F2 expression and gene alteration of E2F2 in LGG was related to low overall survival (consistent with its role as an oncogene), the E2F2 expression and gene alteration of E2F1 showed no significant correlation with OS in the context of GBM. Furthermore, the E2F2 expression was positively related to cancer purity and negatively related to macrophages, neutrophils, and DCs of GBM; however, the E2F2 expression in GBM showed no significant correlation with CD4+ T cells. The E2F2 expression in LGG showed a positive correlation with infiltrating levels of B cells, CD4+ T cells, macrophages, neutrophils, and dendritic cells but not with tumor purity. Specifically, E2F2 was significantly correlated with monocyte markers (CD86), TAM markers (CCL2, IL10), and M2 macrophage markers (VSIG4) in GBM. E2F2 was significantly correlated with monocyte markers (CD86, CD115), TAM markers (CD68, IL10), M1 macrophage markers (IRF5), and M2 macrophage markers (CD163, VSIG4, MS4A4A) in LGG. This suggests that E2F2 plays a role in regulating monocyte polarization in GBM and LGG.

E2F3 overexpression is a cancer suppressor event in many types of tumors, including brain and CNS cancers [32, 40, 41]. miR128-1 inhibited GBM growth by targeting E2F3 [40]. The primary factor of miR-195-mediated cell arrest was E2F3 [41]. Increased expression in E2F3a played a significant role in the development of glioma [2]. Interestingly, SNHG5 promoted the development of glioma by targeting E2F3 [42]. In our study, the E2F3 expression was upregulated in GBM and LGG. The E2F3 expression and gene alteration of E2F3 in LGG and GBM showed no correlation with survival outcomes. However, the E2F3 expression was positively related to tumor purity and neutrophil infiltration remarkably in GBM. About LGG, the E2F3 expression showed a positive correlation with tumor purity and infiltrating levels of B cells, CD8+ T cells, and dendritic cells but not with macrophages and neutrophils.

TFs play a vital role in inhibiting proliferation-related genes. As a member of the TFs E2F family, E2F4 is rich in nonproliferating and differentiated cells [43]. Decreased levels of E2F4 in human GBM cells induced G2/M cell cycle arrest with a concomitant 2-fold decrease with S phase cell [16]. In our study, the expression of E2F4 in GBM and LGG was higher than that in normal tissue. High E2F4 expression in LGG was related to poor overall survival, while the E2F4 expression and gene alteration of E2F4 of GBM showed no correlation with OS. In addition, the E2F4 expression in GBM demonstrated a very weak interrelation with dendritic cell infiltration. E2F4 expression level in LGG was positively related to CD4+ T cells, neutrophil, B cells, macrophage, and DCs. Specifically, E2F4 was significantly correlated with monocyte markers (CD86), TAM markers (CD68), M1 macrophage markers (IRF5), and M2 macrophage markers (CD163, VSIG4, MS4A4A) in LGG. This suggests that E2F4 plays a role in regulating monocyte polarization in LGG.

E2F5 was highly expressed in all kinds of tumors, such as prostate tumor [44] and glioblastoma [45]. In addition, silencing of E2F5 inhibited the proliferation of GBM cells and induced cell cycle arrest. More importantly, reintroduction of E2F5 into GBM cells reversed the tumor-suppressive function of miR-1179 [17]. miRNA-129-3p suppressed growth of glioblastoma via targeting E2F5 [45]. Moreover, Let-7c inhibited glioma development by targeting E2F5 [46]. In our study, the expression of E2F5 in GBM and LGG was higher than that in normal tissue. The E2F5 expression and gene alteration of E2F5 in LGG and GBM showed no correlation with OS. However, the E2F5 expression of GBM was positively related to cancer purity and neutrophil infiltration. The E2F5 expression of LGG showed a positive correlation with tumor purity and infiltrating levels of B cells, CD8+ T cells, and dendritic cells but not with infiltrating level of macrophages. Specifically, E2F5 was significantly correlated with monocyte markers (CD115) and M1 macrophage markers (IRF5) in GBM. This suggests that E2F5 plays a role in regulating monocyte polarization in GBM.

The combination of E2F6 and MATN1-AS1 (which negatively targets RELA) was shown to inhibit the MAPK signaling pathway, thus inhibiting the development of GBM [18]. In our study, the expression of E2F6 in GBM and LGG was higher than that in normal tissue. Increased E2F6 expression and gene alteration of E2F6 in LGG was related to poor OS, while the E2F6 expression of GBM showed no correlation with OS. In addition, the E2F6 expression of GBM was positively related to cancer purity, CD8+ T cells, and macrophages but not with neutrophils. The E2F6 expression in LGG showed a significant positive correlation with tumor purity and infiltrating levels of B cells, CD8+ T cells, macrophages, neutrophils, and dendritic cells but not with CD4+ T cells. Specifically, E2F6 was significantly correlated with monocyte markers (CD86, CD115), TAM markers (CD68, IL10), M1 macrophage markers (IRF5), and M2 macrophage markers (VSIG4, MS4A4A) in GBM. This suggests that E2F6 plays a role in regulating monocyte polarization in GBM.

E2F7 and E2F8 function as transcriptional repressors [47]. E2F7 could induce autophagy of glioma [19]. HOXD-AS1 regulated glioma progress via E2F8 [20]. In our study, the expressions of E2F7 and E2F8 in GBM and LGG were higher than those in normal tissues. High E2F7 and E2F8 expressions and gene alteration of E2F7 and E2F8 in LGG were associated with poor OS. Moreover, the E2F7 and E2F8 expressions were positively related to tumor purity in GBM remarkably. E2F7 expression level in LGG showed a positive correlation with tumor purity and infiltrating levels of B cells, CD8+ T cells, macrophages, and dendritic cells. Similarly, E2F8 expression level in LGG showed a positive correlation with infiltrating levels of B cells, CD8+ T cells, CD4+ T cells, macrophages, neutrophils, and dendritic cells. Specifically, E2F7 was significantly correlated with TAM markers (IL10) in GBM. E2F8 was significantly correlated with M2 macrophage markers (VSIG4) in GBM. E2F8 was significantly correlated with monocyte markers (CD68), TAM markers (CCL2, IL10), M1 macrophage markers (IRF5), and M2 macrophage markers (CD163, MS4A4A) in LGG. This suggests that E2F8 plays a role in regulating monocyte polarization.

## 5. Conclusions

In this study, we systematically analyzed the expressions of E2Fs in brain and CNS cancer tissues, assessed their prognostic value, and investigated their correlation with tumor-infiltrating immune cells. Our findings characterize the heterogeneity and complexity of the molecular biology and the immune infiltrates in brain and CNS cancers. Bioinformatics analysis indicated that the increased expressions of E2F1, 2, and 5–8 in GBM tissues and those of E2F5 and 6 in LGG tissues may play a vital role in oncogenesis. High E2F1–8 expressions may serve as molecular markers to identify high-risk subgroups of patients with GBM. Similarly, high E2F2–8 expressions may help identify high-risk patients in LGG. Our findings suggest that E2F1–8 and E2F2–8 are potential prognostic biomarkers for GBM and LGG, respectively. However, increased expressions of E2F3–6 in GBM correlated with poor prognosis and increased infiltration of CD8+ T cells, macrophages, neutrophils, and DCs. Increased expressions of E2F1-8 in LGG correlated with poor prognosis and increased infiltration of B cells, CD8+ T cells, CD4+ T cells, macrophages, neutrophils, and DCs. Therefore, E2F3–6 and E2F1–8 are potential therapeutic targets in patients with immune cell infiltration of GBM and LGG, respectively.

## Figures and Tables

**Table 1:** The significant changes of the E2F expression in transcription level between different types of brain and CNS cancer and normal brain and CNS tissues (Oncomine database).

	Type of breast cancer versus normal breast tissue	Fold change	*P* value	*t*-test	Source and/or reference
E2F1	NA	NA	NA	NA	NA
E2F2	NA	NA	NA	NA	NA
E2F3	NA	NA	NA	NA	NA
E2F4	NA	NA	NA	NA	NA
E2F5	Anaplastic oligodendroglioma	2.425	3.64*e*−10	9.420	French Brain Statistics [28]
	Anaplastic astrocytoma	2.545	1.45*e*−7	6.181	Sun Brain Statistics [29]
	Oligodendroglioma	2.580	1.99*e*−7	5.707	Sun Brain Statistics [29]
	Glioblastoma	2.877	1.78*e*−9	7.518	Sun Brain Statistics [29]
	Glioblastoma	2.016	1.59*e*−7	10.319	Murat Brain Statistics [30]
E2F6	NA	NA	NA	NA	NA
E2F7	Glioblastoma	4.632	1.08*e*−28	17.833	Murat Brain Statistics [30]
	Glioblastoma	5.823	1.31*e*−21	13.696	Sun Brain Statistics [29]
	Anaplastic oligodendroglioma	2.282	4.46*e*−5	4.698	French Brain Statistics [28]
E2F8	Anaplastic astrocytoma	4.196	8.33*e*−6	4.893	Sun Brain Statistics [29]
	Glioblastoma	7.097	1.25*e*−9	8.349	Sun Brain Statistics [29]
	Oligodendroglioma	3.341	9.18*e*−6	5.003	Sun Brain Statistics [29]

NA: not available; TCGA: The Cancer Genome Atlas.

**Table 2:** Correlation analysis between E2F and relate genes and markers of monocyte, TAM, and macrophages in TIMER.

Description	Gene markers	GBM															
		E2F1		E2F2		E2F3		E2F4		E2F5		E2F6		E2F7		E2F8	
		cor	*P*	cor	*P*	cor	*P*	cor	*P*	cor	*P*	cor	*P*	cor	*P*	cor	*P*

Monocyte	CD86	-0.359	^∗∗∗^	-0.328	^∗∗∗^	-0.242	^∗∗^	-0.140	0.084	-0.261	^∗^	-0.312	^∗∗∗^	-0.193	0.016	-0.224	^∗^
	CD115 (CSF1R)	-0.239	^∗^	-0.227	^∗^	-0.097	0.229	-0.006	0.933	-0.349	^∗∗∗^	-0.352	^∗∗∗^	-0.150	0.062	-0.143	0.076

TAM	CCL2	-0.383	^∗∗∗^	-0.415	^∗∗∗^	-0.309	^∗∗^	-0.111	0.17	-0.203	0.011	-0.228	^∗^	-0.170	0.035	-0.167	0.038
	CD68	-0.246	^∗^	-0.210	^∗^	-0.097	0.228	0.010	0.901	-0.306	^∗∗^	-0.380	^∗∗∗^	-0.180	0.025	-0.164	0.042
	IL10	-0.392	^∗∗∗^	-0.357	^∗∗∗^	-0.289	^∗∗^	-0.141	0.081	-0.259	^∗^	-0.398	^∗∗∗^	-0.326	^∗∗∗^	-0.302	^∗∗^

M1 macrophage	INOS (NOS2)	0.006	0.936	-0.082	0.308	-0.106	0.19	0.164	0.042	-0.114	0.159	0.041	0.61	0.129	0.11	0.116	0.153
	IRF5	-0.246	^∗^	-0.262	^∗^	-0.149	0.065	0.064	0.429	-0.362	^∗∗∗^	-0.395	^∗∗∗^	-0.084	0.296	-0.134	0.098
	COX2 (PTGS2)	-0.182	0.023	-0.140	0.082	0.064	0.431	0.035	0.661	-0.091	0.259	-0.087	0.279	-0.032	0.691	0.005	0.944

M2 macrophage	CD163	-0.205	0.010	-0.234	^∗^	-0.126	0.118	-0.070	0.386	-0.192	0.017	-0.243	^∗^	-0.060	0.458	-0.103	0.203
	VSIG4	-0.389	^∗∗∗^	-0.346	^∗∗∗^	-0.282	^∗∗^	-0.156	0.052	-0.249	^∗^	-0.373	^∗∗∗^	-0.276	^∗∗^	-0.332	^∗∗∗^
	MS4A4A	-0.319	^∗∗∗^	-0.295	^∗∗^	-0.231	^∗^	-0.173	0.031	-0.253	^∗^	-0.313	^∗∗∗^	-0.241	^∗^	-0.233	^∗^

Description	Gene markers	LGG															
		E2F1		E2F2		E2F3		E2F4		E2F5		E2F6		E2F7		E2F8	
		cor	*P*	cor	*P*	cor	*P*	cor	*P*	cor	*P*	cor	*P*	cor	*P*	cor	*P*

Monocyte	CD86	-0.203	^∗∗∗^	0.228	^∗∗∗^	0.061	0.166	0.185	^∗∗∗^	0.004	0.910	0.074	0.089	-0.004	0.921	0.157	^∗∗^
	CD115 (CSF1R)	-0.285	^∗∗∗^	0.209	^∗∗∗^	0.048	0.269	0.114	^∗^	-0.029	0.510	-0.041	0.343	-0.147	^∗∗^	0.055	0.208

TAM	CCL2	-0.19	^∗∗∗^	0.065	0.137	-0.027	0.532	0.096	0.028	-0.057	0.188	-0.004	0.922	0.01	0.818	0.062	0.153
	CD68	-0.124	^∗^	0.28	^∗∗∗^	0.076	0.084	0.267	^∗∗∗^	0.057	0.192	0.103	0.018	0.098	0.025	0.224	^∗∗∗^
	IL10	-0.152	^∗∗^	0.183	^∗∗∗^	0.043	0.321	0.168	^∗∗^	0.002	0.954	0.035	0.421	0.049	0.257	0.145	^∗∗^

M1 macrophage	INOS (NOS2)	0.197	^∗∗∗^	-0.005	0.905	0.011	0.801	0.012	0.771	-0.059	0.178	0.014	0.738	0.093	0.034	0.073	0.093
	IRF5	-0.117	^∗^	0.226	^∗∗∗^	0.008	0.842	0.285	^∗∗∗^	-0.076	0.081	0.038	0.386	0.05	0.250	0.183	^∗∗∗^
	COX2 (PTGS2)	0.009	0.830	0.008	0.849	0.077	0.079	-0.038	0.386	-0.109	0.012	-0.072	0.100	0.047	0.286	0.051	0.242

M2 macrophage	CD163	-0.077	0.077	0.224	^∗∗∗^	0.131	^∗^	0.193	^∗∗∗^	0.023	0.597	0.153	^∗∗^	0.142	^∗^	0.203	^∗∗∗^
	VSIG4	-0.255	^∗∗∗^	0.256	^∗∗∗^	0.098	0.024	0.183	^∗∗∗^	0.041	0.349	0.08	0.067	-0.079	0.072	0.107	0.014
	MS4A4A	-0.157	^∗∗^	0.28	^∗∗∗^	0.134	^∗^	0.193	^∗∗∗^	0.104	0.017	0.145	^∗∗^	0.07	0.107	0.212	^∗∗∗^

Cor: *R* value of Spearman's correlation; TAM: tumor-associated macrophages. ^∗^*P* < 0.01; ^∗∗^*P* < 0.001; ^∗∗∗^*P* < 0.0001.

## Data Availability

The data used to support the findings of this study are included within the article.

## Abbreviations

E2Fs: E2F transcription factors
CNS: Central nervous system
GBM: Glioblastoma multiforme
LGG: Low grade glioma
PPI: Protein-protein interaction
OS: Overall survival
TAM: Tumor-associated macrophages
GO: Gene ontology
KEGG: Kyoto Encyclopedia of Genes and Genomes.
